# ABCG2 is not able to catalyze glutathione efflux and does not contribute to GSH-dependent collateral sensitivity

**DOI:** 10.3389/fphar.2013.00138

**Published:** 2013-11-07

**Authors:** Charlotte Gauthier, Csilla Ozvegy-Laczka, Gergely Szakacs, Balazs Sarkadi, Attilio Di Pietro

**Affiliations:** ^1^Drug Resistance Mechanism and Modulation Group, Ligue 2013 Certified, Bases Moléculaires et Structurales des Systèmes Infectieux, UMR5086, Centre National de la Recherche Scientifique, Université de Lyon, Institut de Biologie et Chimie des Protéines, University of LyonLyon, France; ^2^Research Group of Active Transport Proteins, Institute of Enzymology, Research Centre for Natural Sciences, Hungarian Academy of SciencesBudapest, Hungary; ^3^Institute of Enzymology, Research Centre for Natural Sciences, Hungarian Academy of SciencesBudapest, Hungary; ^4^Molecular Biophysics Research Group, Hungarian Academy of Sciences, Semmelweiss UniversityBudapest, Hungary

**Keywords:** breast cancer resistance protein ABCG2, collateral sensitivity, glutathione efflux, intracellular glutathione depletion, multidrug resistance protein ABCC1, selective apoptosis, modulators

## Abstract

ABCG2 is a key human ATP-binding cassette (ABC) transporter mediating cancer cell chemoresistance. In the case of ABCC1, another multidrug transporter, earlier findings documented that certain modulators greatly increase ABCC1-mediated glutathione (GSH) efflux and, upon depletion of intracellular GSH, induce “collateral sensitivity” leading to the apoptosis of multidrug resistant cells. Recently, it has been suggested that ABCG2 may mediate an active GSH transport. In order to explore if ABCG2-overexpressing cells may be similarly targeted, we first looked for the effects of ABCG2 expression on cellular GSH levels, and for an ABCG2-dependent GSH transport in HEK293 and MCF7 cells. We found that, while ABCG2 overexpression altered intracellular GSH levels in these transfected or drug-selected cells, ABCG2 inhibitors or transport modulators did not influence GSH efflux. We then performed direct measurements of drug-stimulated ATPase activity and ^3^H-GSH transport in inside-out membrane vesicles of human ABC transporter-overexpressing Sf9 insect cells. Our results indicate that ABCG2-ATPase is not modulated by GSH and, in contrast to ABCC1, ABCG2 does not catalyze any significant GSH transport. Our data suggest no direct interaction between the ABCG2 transporter and GSH, although a long-term modulation of cellular GSH by ABCG2 cannot be excluded.

## INTRODUCTION

The development of multidrug resistance (MDR) constitutes a major issue in cancer treatment. Overexpression of the three human ATP-binding cassette (ABC) transporters, ABCB1 (P-glycoprotein/P-gp; Juliano and Ling, 1976), ABCC1 (multidrug resistance protein 1/MRP1; Cole et al., 1992), and ABCG2 (breast cancer resistance protein, BCRP; Allikmets et al., 1998; Doyle et al., 1998; Miyake et al., 1999) has been proposed as one of the main causes of the MDR phenotype in resistant cancer cells. These proteins use ATP hydrolysis as energy source to catalyze the efflux of multiple structurally and functionally diverse chemotherapeutics from cancer cells.

Research has mainly focused on inhibitors development, in order to block this efflux mechanism and then restore chemotherapeutics efficacy. Unfortunately, scientists had to face clinical failures of third-generation ABCB1 inhibitors optimized *in vitro*, such as Zosuquidar (Cripe et al., 2010) or valspodar (Kolitz et al., 2010). In addition to improving clinical trials conducted with efflux inhibitors (Robey et al., 2010), alternative strategies to overcome the MDR phenotype need to be explored. Recently, a new strategy, so called collateral sensitivity (CS), characterized by hypersensitivity to small molecules triggering a preferential cytotoxicity, has been studied (Szakacs et al., 2006; Hall et al., 2009), and four different mechanisms have been hypothesized to underlie the hypersensitivity of ABCB1- and ABCC1-overexpressing cancer cells. CS agents may (i) produce reactive oxygen species by depleting intracellular ATP; (ii) exploit energetic sensitivities caused by ATP depletion; (iii) induce the extrusion of vital endogenous substrates; or (iv) perturb the plasma membrane (Pluchino et al., 2012).

A screening study identified two compounds as potential ABCG2-related CS agents in HEK293 transfected cells, one of them (NSC103054) directly interacting with the transporter (Deeken et al., 2009), and very recently an ABCG2 inhibitor (NP-1250) was reported to induce CS in mitoxantrone-selected MCF7 cancer cells (Ito et al., 2013). Although a mechanism based on extracellular vesicles photodestruction have been shown for another ABCG2-dependent CS (Goler-Baron and Assaraf, 2012), no direct mechanisms have yet been demonstrated; however, these different studies indicate that CS agents, specific for ABCG2, could be developed.

Reduced glutathione (GSH, γ-glutamyl-cysteinyl-glycine) is a tripeptide ubiquitously expressed in cells and involved in many signaling pathways. It has been shown that ABCC1-overexpressing cells were hypersensitive to verapamil through a sharp GSH depletion due to an ABCC1-mediated efflux (Trompier et al., 2004). This phenomenon was further investigated in order to target resistant cancer cells in the frame of a new strategy to overcome the MDR phenotype in cancer (Barattin et al., 2010; Genoux-Bastide et al., 2011). ABCC1 is also known to transport oxidized glutathione disulfide (GSSG) which is however present in low amounts (Keppler et al., 1997). Based on our experience with ABCC1-specific CS and on recent reports in which ABCG2 was proposed as a new GSH transporter (Brechbuhl et al., 2009, 2010) we aimed at developing new ABCG2-specific modulators able to induce ABCG2-mediated GSH extrusion in order to induce a drastic intracellular GSH depletion leading to cell death.

In this study, we focused on searching inducers of ABCG2-dependent depletion of intracellular GSH among known death inducers of ABCC1-overexpressing cells, such as verapamil and xanthones (Genoux-Bastide et al., 2011), or known ABCG2 inhibitors (Ahmed-Belkacem et al., 2005; Valdameri et al., 2012). To ascertain the direct role of ABCG2 in GSH efflux, we measured direct transport of radioactive GSH in membrane vesicles.

## MATERIALS AND METHODS

### COMPOUNDS

Verapamil, Ko143, apigenin, ATP, chrysin, ditiothreitol (DTT), galangin, 5,5′-dithiobis(2-nitrobenzoic acid; DTNB), β-nicotinamide adenine dinucleotide 2′-phosphate reduced tetrasodium salt hydrate (NADPH), quercetin, glutathione (GSH), glutathione reductase, and bicinchoninic acid (BCA) were purchased from Sigma Aldrich (Saint-Quentin Fallavier, France). Acivicin was purchased from CliniSciences (Montrouge, France). ^3^H-methotrexate and ^3^H-GSH were purchased from Moravek Biochemicals and PerkinElmer, respectively. All other tested compounds were kindly provided by Prof. Ahcène Boumendjel (UJF Grenoble, France) and prepared as previously described (Genoux-Bastide et al., 2011; Valdameri et al., 2012). Tested compounds were dissolved in DMSO and stored at -20°C; they were warmed to 25°C and diluted in Dulbecco’s modified Eagle’s medium (DMEM) just before use (0.5% DMSO final concentration).

### CELL CULTURE

The cell lines were kindly provided by Drs Susan Bates and Robert Robey, NCI, Bethesda, MD, USA. The selected human breast cancer cell line (MCF7-MX100) and the human fibroblast HEK293 cell line transfected with either *ABCG2* (HEK-*ABCG2*) or the empty vector (HEK-pcDNA3.1) were prepared as respectively reported (Honjo et al., 2001; Robey et al., 2003). The HEK293 and MCF7 cells were maintained in DMEM (high glucose, PAA) and in Roswell Park Memorial Institute medium (RPMI-1640, PAA) respectively, supplemented with 10% fetal bovine serum (FBS, PAA), 1% penicillin/streptomycin (PAA) and with 0.75 mg/ml G418 (for HEK-pcDNA3.1 and HEK-*ABCG2* cells) or 100 nM mitoxantrone (for MCF7-MX100 cells). Cells were cultured at 37°C, 5% CO_2_ in a humid atmosphere. Sf9 insect cells were cultured at 27°C in TNM-FH insect medium supplemented with 10% fetal calf serum (FCS) and penicillin (100 U/ml)–streptomycin (100 μg/ml; Sigma Aldrich, Hungary).

### INTRACELLULAR GLUTATHIONE ASSAY

HEK293 and MCF7 cells were seeded in 96-well plates at respective densities of 1 × 10^4^ and 2 × 10^4^ cells/well. After 24 h in culture, cells were exposed to the different compounds during 6 or 24 h under normal culture conditions. They were then washed with 200 μl PBS 1X (PAA), stirred during 1 h at 4°C with 100 μl of 10 mM HCl and freezed at -20°C overnight, to be lysed. The intracellular total glutathione (reduced GSH and oxidized GSSG) was measured using the method described by Tietze (1969) as modified by Anderson (1985). About 70 μl of the lysate were used to measure intracellular total glutathione and 20 μl for protein quantitation, both being performed in 96-well plates. Total glutathione was assessed by adding 100 μl of a reaction buffer containing 266 μM NADPH, *GSH reductase* at 10 U/ml and 555 μM DTNB, and the absorbance was read at 412 nm in a microplate reader (PowerWave 340, Biotek) every 30 s during 2 min. The slope for each sample and glutathione standard range was determined to quantify sample glutathione. Protein quantitation was performed using the BCA assay. The results were expressed in nmol glutathione/mg protein and intracellular total glutathione percentages were calculated using the 0 μM samples as 100%.

### EXTRACELLULAR GLUTATHIONE ASSAY

HEK293 cells were seeded in 24-well plates at a density of 1.5 × 10^5^ cells/well. After 24 h in culture, cells were co-treated with the compound and 0.5 mM acivicin (to block GSH degradation out of the cells) during the 24-h incubation time. Supernatants were collected and cells were washed with 200 μl PBS 1× and treated as for intracellular total glutathione measurement. About 70 μl of the supernatant were used to assess total extracellular glutathione, and protein titration was performed with cell lysate, by the same method as described for intracellular glutathione measurement.

### CELL PROLIFERATION AS DETERMINED BY MTT ASSAY

The MTT colorimetric assay, as previously described (Mosmann, 1983), was used to assess the sensitivity of cells to compounds toxicity. HEK293 cells were seeded in 96-well plates at a density of 1 × 10^4^ cells/well. After 24 h under normal culture conditions, cells were treated with compounds at increasing concentrations. After 72-h incubation under normal culture conditions a 3-(4,5-dimethyl-2-thiazoyl)-2,5-diphenyl-2*H*-tetrazolium Bromide (MTT) solution was added (0.5 mg/ml final concentration) in wells, and cells were incubated for 4 h at 37°C. Thereafter, supernatants were carefully withdrawn and 100 μl/well of the buffer ethanol/DMSO (50/50, v/v) were added to solubilize the reduced formazan dye under stirring. Absorbance at 570 and 690 nm were determined by using a microplate reader (PowerWave 340, Biotek). Results were expressed as the difference between OD_570_ and OD_690_; cell survival percentage was calculated using 0 μM sample OD as 100%.

### MEMBRANE PREPARATION

For obtaining membrane vesicles insect cells were infected with recombinant baculoviruses containing the cDNA of wtABCG2 or ABCG2-K86M (Ozvegy-Laczka et al., 2005) or of ABCC1 (Bakos et al., 1996). Membrane preparation and cholesterol enrichment of ABCG2-containing membranes was then performed as described earlier (Ozvegy et al., 2001; Telbisz et al., 2007).

### ATPase ACTIVITY ASSAY

The ATP hydrolytic activity of ABCG2 has been determined as described in Ozvegy et al. (2001) and Telbisz et al. (2007). When the effect of GSH was investigated a minor modification in the assay buffer was introduced. About 10 mM DTT was used instead of 2 mM to prevent the oxidation of GSH.

### ^3^H-METHOTREXATE AND ^3^H-GSH TRANSPORT ASSAY

Sf9 membrane vesicles containing 90 μg protein were incubated in the presence or absence of 4 mM MgATP (or 4 mM MgATP + 1 μM Ko143 or 4 μM MK571) in a buffer containing 40 mM 3-(*N*-morpholino) propanesulfonic acid–Tris (pH 7.0), 56 mM KCl, 6 mM MgCl_2_, and 10 mM DTT, in a final volume of 140 μl, at 37°C for 5–10 min as indicated on the figure legends. The measurement was started by the addition of 50 μM [^3^H]methotrexate (Moravek Biochemicals) or 0.1–1 mM ^3^H-GSH and carried out as described earlier (Ozvegy-Laczka et al., 2005).

### STATISTICAL ANALYSIS

Statistic *t*-test analyses were performed using the SigmaPlot 12 software with *p < 0.05, **p < 0.01, ***p < 0.001.

## RESULTS

### INTRACELLULAR GLUTATHIONE CONCENTRATION IN ABCG2-OVEREXPRESSING CELLS

**FIGURE 1:**
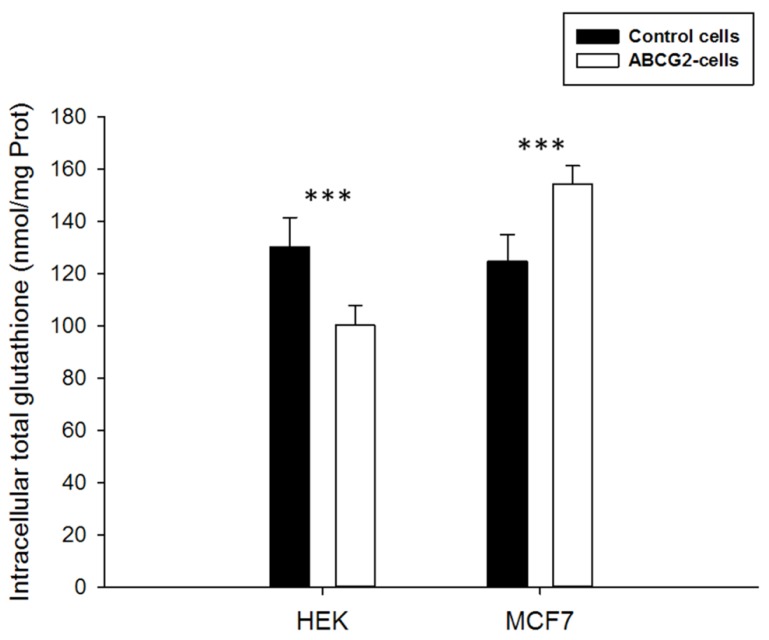
**Basal total intracellular glutathione levels in HEK293 or MCF7 cells.** The values of either ABCG2-overexpressing cells (white bars) or control cells (black bars) represent means ± SD corresponding to at least three independent experiments performed in triplicates. The differences observed between both cell line pairs were significantly different. *t*-test analysis: ***p < 0.001.

In order to determine the influence of ABCG2 on cellular glutathione levels, we used two different cell lines overexpressing this transporter. The high level of ABCG2 expression and functionality, through ability to transport a number of substrate drugs, were previously described, in both transfected HEK-ABCG2 cells (Robey et al., 2003) and drug-selected MCF7-MX100 cancer cells (Honjo et al., 2001). Moreover, we performed western blot analyses which revealed that all cell lines did not express the ABCC1 protein (data not shown). The intracellular concentration of total glutathione (free GSH + oxidized GSSG) appeared to be significantly modulated by the presence of overexpressed ABCG2 (**Figure 1**). The glutathione level was lower in ABCG2-transfected HEK293 cells by comparison to the same cells transfected by the pcDNA3.1 empty vector (100 ± 8 versus 130 ± 11 nmol glutathione/mg protein). Interestingly, in drug-selected MCF7 cancer cells, which also overexpress ABCG2, the intracellular glutathione content was significantly higher than in the parental MCF7 cells (154 ± 7 versus 125 ± 10 nmol glutathione/mg protein). These data may indicate a long-term modulation of glutathione levels in various ABCG2-overexpressing cell types. Since total glutathione is known to be essentially constituted of free GSH and low amounts of oxidized GSSG, we measured both components separately in the different cell lines, upon incubation with 2-vinylpiridine behaving as a thiol scavenger. In all cases, the remaining oxidized GSSG was too low to be detected (not shown here), indicating no evidence of any change in the ratio between reduced and oxidized forms of glutathione.

### INABILITY OF MODULATORS TO STIMULATE AN ACTIVE ABCG2-MEDIATED GLUTATHIONE EFFLUX

**FIGURE 2:**
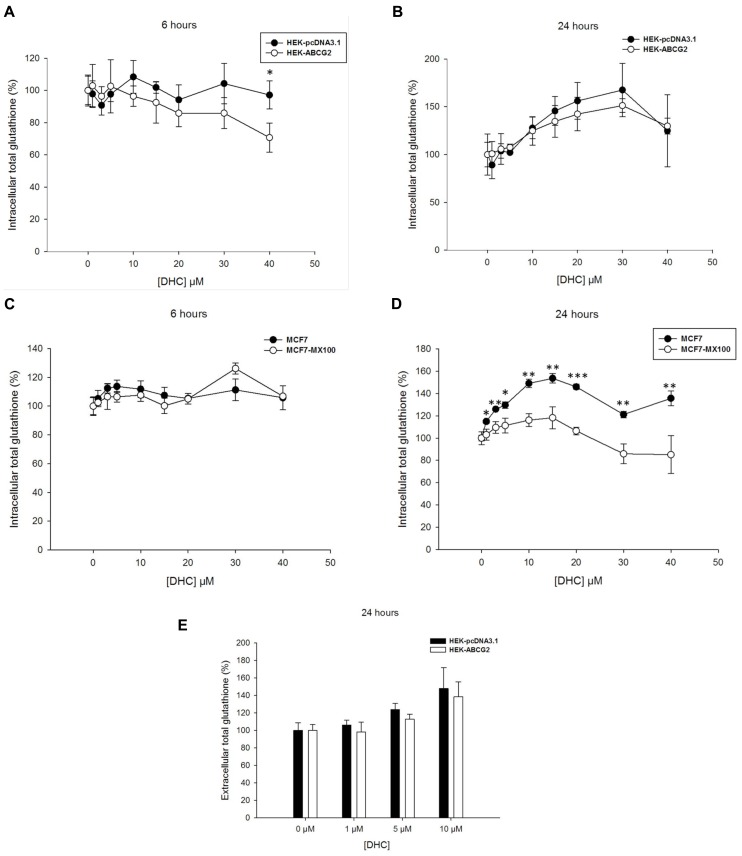
**Effects of 2′,5′-DHC increasing concentrations on total intracellular and extracellular glutathione levels.** 2′,5′-DHC did not induce intracellular GSH depletion in ABCG2 cells (white circles) by comparison to control cells (black circles) in either HEK293 transfected cells during 6 **(A)** or 24 h **(B)**, or MCF7 cancer cells during 6 **(C)** or 24 h **(D)**. Moreover, there was no net ABCG2-dependent increase in extracellular GSH **(E)** induced by 2′,5′-DHC when comparing HEK-ABCG2 (white bars) and HEK-pcDNA3.1 (black bars) cells after 24-h incubation. The values represent means ± SD corresponding to at least two independent experiments performed in triplicates. Only the differences observed in **(D)**, between MCF7 and MCF7-MX100 cell lines at 24 h, were significant. *t*-test analysis: *p < 0.05, **p < 0.01, and ***p < 0.001.

Since the 2′,5′-DHC chalcone was reported to stimulate ABCG2-dependent GSH efflux (Brechbuhl et al., 2010), the effects produced by addition of 2′,5′-DHC at increasing concentrations (up to 40 μM) were analyzed here on the intracellular glutathione levels of both transfected and drug-selected cells. A weak concentration-dependent decrease appeared in ABCG2-transfected cells after 6-h incubation with 2′,5′-DHC (**Figure 2A**), but not after 24-h incubation where an increase in intracellular glutathione content was observed in both cell lines (**Figure 2B**). By contrast, in drug-selected MCF7 cells, no decrease in glutathione content appeared after 6-h incubation (**Figure 2C**); a significant difference in glutathione level was observed after 24-h incubation, which was however essentially due to a higher increase in control cells than in ABCG2-overexpressing cells (**Figure 2D**). The extracellular glutathione content increased after 24-h incubation of ABCG2-transfected cells with increasing 2′,5′-DHC concentrations (around 40% at 10 μM), but the increase was at least as high in control cells indicating that it was not dependent on ABCG2 (**Figure 2E**).

**FIGURE 3:**
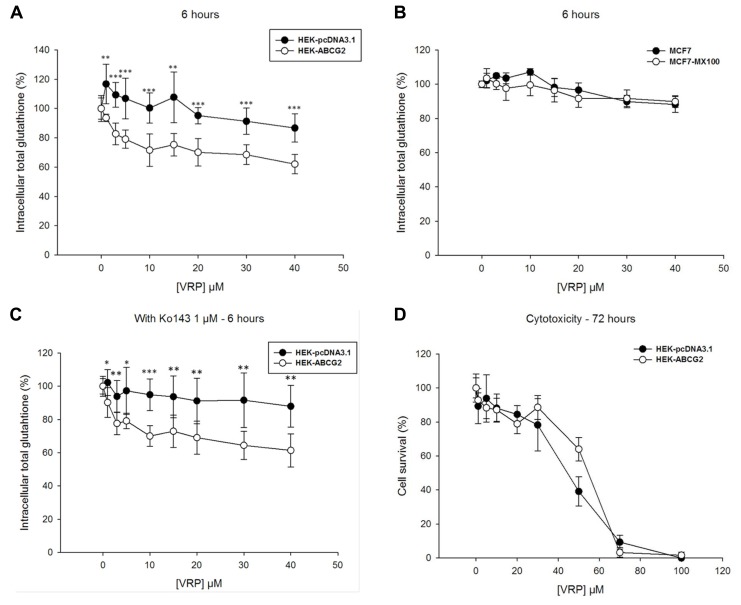
**Effects of verapamil on intracellular glutathione depletion and cells survival.** Verapamil induced a significant intracellular GSH depletion in HEK293 transfected cells during 6-h incubation **(A)**, but not in the MCF7 selected cells **(B)** when comparing ABCG2-overexpressing cells (white circles) to control cells (black circles). This weak effect was not inhibited by Ko143 **(C)**, the difference in intracellular glutathione remaining unchanged. It was not either correlated to any ABCG2-specific collateral sensitivity in MTT cell survival assays **(D)** with ABCG2-overexpressing cells (white circles) and control cells (black circles). The values represent means ± SD corresponding to at least two independent experiments performed in triplicates. *t*-test analysis: *p < 0.05, **p < 0.01, and ***p < 0.001.

We then studied the effects of verapamil which is known to strongly stimulate GSH efflux in ABCC1-overexpressing cells, leading to a fast and massive intracellular glutathione depletion able to trigger apoptosis (Trompier et al., 2004; Perrotton et al., 2007). A significant decrease of intracellular glutathione was indeed observed in ABCG2-transfected cells with increasing verapamil concentrations, up to 40 μM, which was 25–30% higher than in control HEK293 cells (**Figure 3A**). However, no decrease in glutathione content was observed under the same conditions with the ABCG2-overexpressing drug-selected cells, which behaved similarly to control MCF7 cells (**Figure 3B**). The ABCG2-related decrease of intracellular glutathione was therefore further characterized in the presence of Ko143, a potent and specific inhibitor of ABCG2 transport activity. **Figure 3C** shows no significant alteration by comparison to **Figure 3A**, therefore indicating that such a decrease in intracellular glutathione was not dependent on ABCG2 activity. This was further confirmed by the absence of any CS toward verapamil cytotoxicity, as determined by MTT assays, since the ABCG2-transfected cells were not more sensitive than the control cells (**Figure 3D**).

**FIGURE 4:**
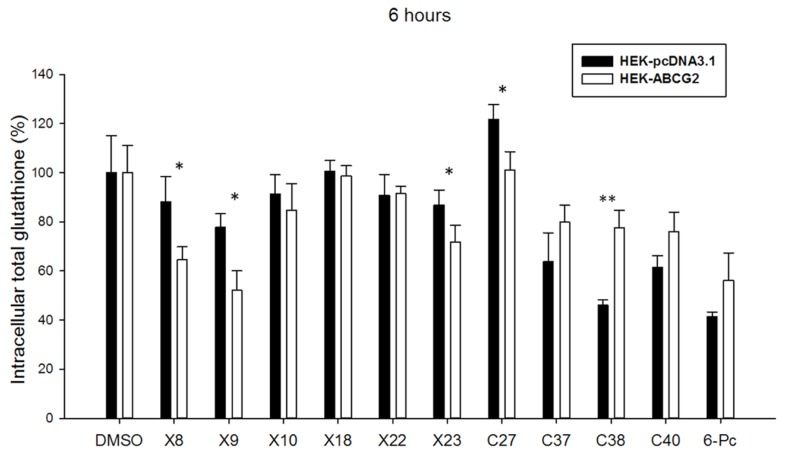
**Effects of other modulators on ABGC2-specific intracellular glutathione depletion.** Screening of Xanthones (Genoux-Bastide et al., 2011), Chalcones (Valdameri et al., 2012), and 6-Prenylchrysin (Ahmed-Belkacem et al., 2005) reveal a significant ABCG2-dependent intracellular glutathione depletion in 6-h incubation for some compounds, as indicated. The values represent means ± SD corresponding to at least two independent experiments performed in triplicates. *t*-test analysis: *p < 0.05, **p < 0.01.

Finally, two other series of compounds were investigated for their ability to modify the intracellular glutathione level. The first series included xanthones (X8, 9, 10, 18, 22, 23) known to induce, similarly as verapamil, a strong depletion in intracellular glutathione in ABCC1-overexpressing cells (Genoux-Bastide et al., 2011), and the second series contained chalcones (C27, 37, 38, 40; Valdameri et al., 2012) and 6-prenylchrysin (6-Pc; Ahmed-Belkacem et al., 2005) known as ABCG2 inhibitors. **Figure 4** shows that some xanthones induced a significant decrease in intracellular glutathione, up to around 30% for X8 and X9 and 20% for X23, similarly to the effect observed with verapamil in **Figure 3A**. By contrast, the ABCG2 inhibitory chalcones, except for C27, and 6-prenylchrysin did not induce any decrease of intracellular glutathione in ABCG2-transfected cells.

### NO DETECTABLE INTERACTION BETWEEN GSH AND ABCG2 IN EITHER ATPase OR TRANSPORT ASSAY

**FIGURE 5:**
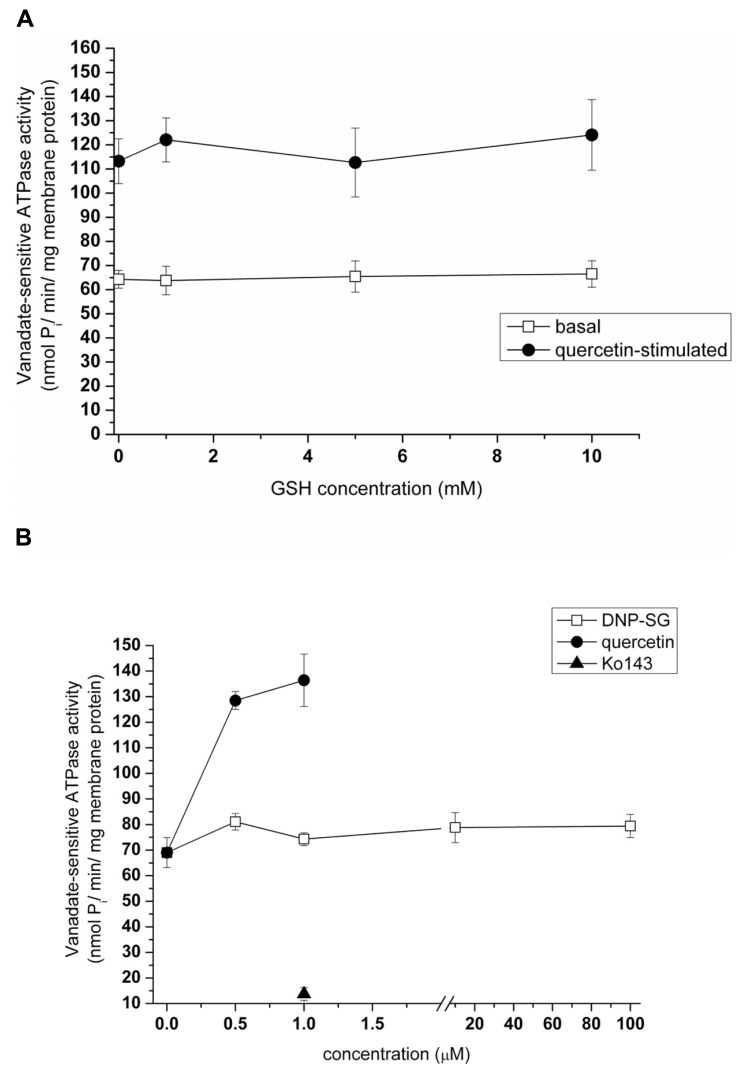
**Effects of GSH and DNP-SG on the ATP hydrolytic activity of ABCG2.** Sodium orthovanadate-dependent ATP cleavage by wtABCG2 in 2 mM cholesterol-loaded Sf9 membrane vesicles was determined in the presence of increasing concentrations of GSH **(A)** both in the absence (“basal”) and in the presence of a known transported substrate (1 μM quercetin, “quercetin-stimulated”) without producing any significant effect. It was also assayed with increasing concentrations of DNP-SG or quercetin or with 1 μM Ko143 **(B)**. Data points represent the average ± SD values of two independent measurements.

We previously demonstrated that the baculovirus-insect cell heterologous expression system is a useful tool for the detection of interactions between a given test compound and ABCG2 (Szakács et al., 2008). Briefly, compounds modifying the ATP hydrolytic activity of ABCG2 interact with the transporter, and can be either transported substrates or inhibitors of the protein. In order to define whether GSH is able to interact with ABCG2, we have tested its effect in the ATPase assay using cholesterol-loaded Sf9 vesicles ensuring higher ABCG2 activity. We found that the ATPase activity of ABCG2 was not affected by GSH addition up to 10 mM, by contrast to a transported substrate such as 1 μM quercetin which stimulated twofold the basal ATPase activity, and the ABCG2-specific inhibitor Ko143 which fully inhibited (**Figures 5A, B**). GSH did not alter the quercetin-stimulated ATPase activity either. Moreover, no effect was produced by the glutathione-conjugate *S*-(2,4-dinitrophenyl)glutathione (DNP-SG; **Figure 5B**) known to be actively transported by ABCC1 (Leier et al., 1994).

**FIGURE 6:**
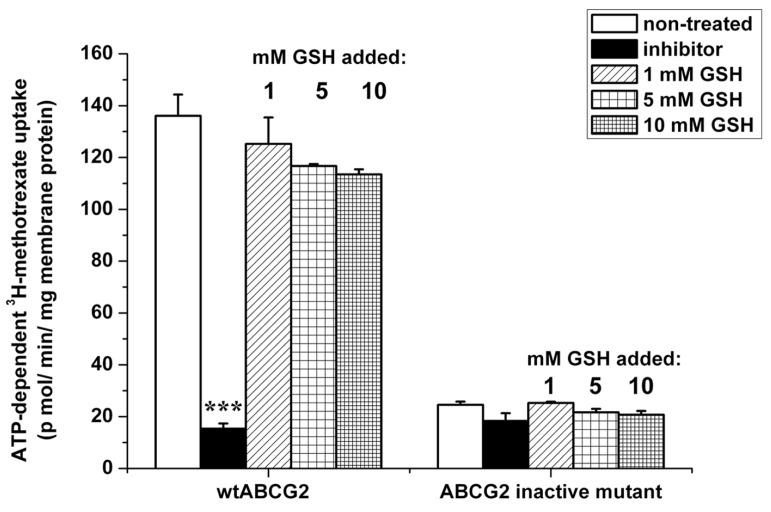
**Effect of GSH on the transport of methotrexate by ABCG2.** ATP-dependent transport of ^3^H-methotrexate in 2 mM cholesterol-loaded insect-cell membranes expressing ABCG2 (either wild-type or the inactive K86M mutant) was measured for 10 min at 37°C. Transport was determined in the absence or presence of an ABCG2-specific inhibitor (1 μM Ko143) or 1–10 mM GSH. Bars represent the average ± SD values of at least two measurements. *t*-test analysis: ***p < 0.001.

As the ATPase assay did not give any proof of interaction between GSH and ABCG2, we investigated the ability of GSH to modify the transport of ^3^H-methotrexate. As shown in **Figure 6**, the ABCG2-mediated transport of tritiated methotrexate was not significantly inhibited by GSH addition, up to a 10 mM concentration, by difference with 1 μM Ko143 leading to the low background level observed with inactive mutant ABCG2. This contrasts with the reported prevention by 10 μM methotrexate against the increased extracellular GSH level observed in transformed yeast expressing human ABCG2 (Brechbuhl et al., 2010).

### INABILITY OF ABCG2 TO CATALYZE AN ACTIVE TRANSPORT OF GSH

**FIGURE 7:**
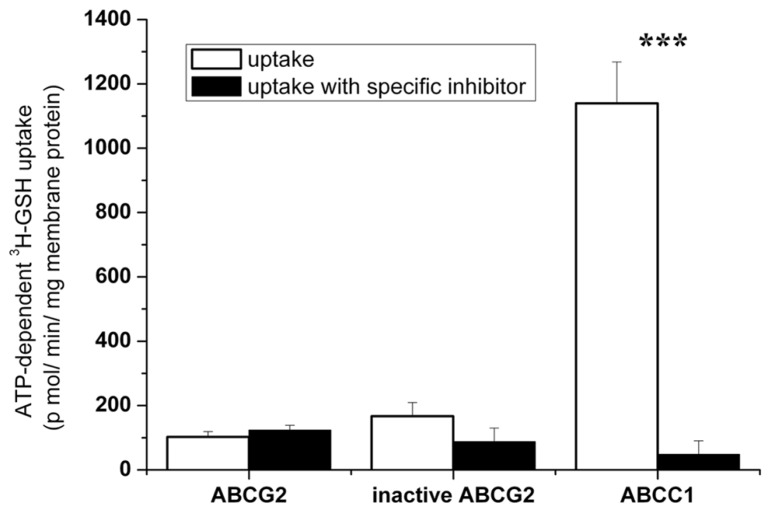
**Investigation of the transport of ^**3**^H-GSH into wtABCG2-containing insect vesicles.** Uptake of 500 μM ^3^H-GSH was measured in Sf9 vesicles expressing ABCC1, wtABCG2, or inactive ABCG2, for 5 min at 37°C. ATP-dependent transport with or without the ABCC1- or ABCG2-specific inhibitor, 4 μM MK-571 or 1 μM Ko143 respectively, is shown. Bars represent the average of three independent measurements ± SD values. *t*-test analysis: ***p < 0.001.

Finally, we measured the direct transport of ^3^H-GSH into ABCG2-containing membrane vesicles. We found that, in contrast to ABCC1 serving as a positive control, no direct, ATP-dependent and specific inhibitor-sensitive, transport of tritiated GSH by ABCG2 could be detected in insect-cell membrane vesicles (**Figure 7**). Any ABCG2-mediated GSH transport could not be either determined at other ^3^H-GSH concentrations (0.1 or 1 mM, data not shown).

## DISCUSSION

The key results of this paper strongly suggest that human ABCG2 is unable to transport GSH. This has been demonstrated by direct measurement of ATP-dependent tritiated GSH uptake in inverted vesicles of insect-cell membranes overexpressing human ABCG2. In contrast, human ABCC1 catalyzed a high level of ATP-dependent and MK571-sensitive GSH transport under the same conditions. There was a low level of GSH accumulation in the presence of ABCG2 observed without ATP, which was also observed in the presence of the selective ABCG2 inhibitor Ko143 (Allen et al., 2002), or when the catalytically inactive K86M ABCG2 mutant was expressed. Thus, this background GSH binding could not be attributed to any ABCG2-mediated active transport.

This result is fully consistent with the lack of effect of GSH, even at high concentrations, on both basal and quercetin-stimulated ABCG2-ATPase activity of the insect cell membrane vesicles. Indeed, transported substrates such as prazosin, quercetin, or nilotinib (Telbisz et al., 2012) strongly stimulate the basal ATPase activity, then enhancing “coupled” ATPase activity. Our present results also show the lack of any effect by DNP-SG on the ABCG2 transporter, suggesting that glutathione conjugates are not transported by ABCG2. This is in contrast to various compounds conjugated with either sulfate (Suzuki et al., 2003) or glucuronate (Chen et al., 2003), whereas DNP-SG is actively transported by ABCC1 (Leier et al., 1994). The lack of ABCG2-mediated GSH transport is also consistent with the lack of any antagonism by GSH addition against ABCG2-mediated tritiated-methotrexate transport in inverted vesicles. These results, however disagree with the methotrexate-induced inhibition of GSH efflux reported in transformed yeast cells, expressing human ABCG2 (Brechbuhl et al., 2010).

Our results from experiments using membrane vesicles are quite consistent with those obtained with either transfected or drug-selected ABCG2-overexpressing cells where we did not observe any sharp and rapid decrease of intracellular GSH stimulable by modulators (such as 2′,5′-DHC, verapamil, or xanthones), or alterable by ABCG2 inactivation (such as using the potent Ko143 inhibitor). In addition, there was no inverse correlation between the observed decrease of intracellular GSH and increase of extracellular GSH, as also noticed in other drug-selected cancer cells overexpressing ABCG2 (Brechbuhl et al., 2010). This contrasts with the strong effects clearly observed with ABCC1-overexpressing cells (Trompier et al., 2004; Perrotton et al., 2007; Barattin et al., 2010; Genoux-Bastide et al., 2011).

Nevertheless, the intracellular total glutathione concentration appeared to be modulated by overexpressed ABCG2 since, for unknown reasons possibly resulting from different signaling pathways, glutathione was lower in HEK293 transfected cells and higher in the drug-selected MCF7 cancer cells by comparison to their respective control cells. In addition, a significant decrease of intracellular glutathione was actually observed, either in some cases with 2′,5′-DHC, as previously reported (Brechbuhl et al., 2010), or with known ABCC1 modulators such as verapamil and xanthones. Such a decrease of intracellular glutathione however displayed special characteristics, such as being slow, requiring at least 6–24 h incubation, and not depending on ABCG2 activity since it was not altered by Ko143 inhibition. These results are more likely compatible with the induction of associated signaling pathways, leading to changes in intracellular GSH, than with a direct GSH transport.

ABCG2 is known to be regulated by a number of signaling pathways including NF-KB (Shen et al., 2010), RAR/RXR (Hessel and Lampen, 2010), hedgehog (Singh et al., 2011), P13K/AKT (Nakanishi and Ross, 2012), JNK1/c-jun (Zhu et al., 2012), HER2 and EGFR/HER1 (Gilani et al., 2012), and ERK1/2 (de Boussac et al., 2012). Some signaling pathways, such as notch (Battacharya et al., 2007), CXCL12-CXCR4 (Katoh and Katoh, 2010), Oct4-TCL1-AKT (Wang et al., 2010), PTEN/P13K/Akt (Li et al., 2011), β-catenin/Tcf (Usongo and Farookhi, 2012), AhR (Dubrovska et al., 2012), and HIF-2α with TGF-β/Smad2 (Cui et al., 2013) are related to the ABCG2 status as a marker of stem cells or stem-like cancer cells. Nrf2, a critical transcription factor that regulates antioxidants, detoxification enzymes, and drug efflux proteins in response to oxidative stress (Hong et al., 2010; Singh et al., 2010; Chen et al., 2012; Zhang et al., 2012; Ishikawa et al., 2013; Shelton and Jaiswal, 2013), may provide a link between cellular GSH homeostasis and ABCG2 expression. Whatever the mechanism(s) involved, changes in intracellular GSH are evidently too slow and too low to induce a sufficient cellular GSH depletion susceptible to trigger CS-induced cell apoptosis, as observed for ABCC1. It is still an important question if, and how, an ABCG2-dependent CS can effectively be produced. Indeed, the few known examples report very low selectivity ratio values, limited to 2.5–3 (Deeken et al., 2009), by comparison with the values, at least one order of magnitude higher, reported for both ABCB1- (Ludwig et al., 2006; Hall et al., 2009; Türk et al., 2009; Pluchino et al., 2012) and ABCC1- (Trompier et al., 2004; Barattin et al., 2010; Genoux-Bastide et al., 2011) overexpressing cells. Such a difference may be at least partly related to the complex involvement of ABCG2 in many signaling pathways. Further identification and characterization of mechanisms directly connecting ABCG2 to CS-associated apoptosis and signaling are mandatory for establishing a new therapeutic strategy, selectively targeting and eliminating resistant cancer cells.

## Conflict of Interest Statement

The authors declare that the research was conducted in the absence of any commercial or financial relationships that could be construed as a potential conflict of interest.

## Abbreviations

ABC: ATP-binding cassette
BCRP (ABCG2): breast cancer resistance protein
CS: collateral sensitivity
2′,5′-DHC: 2′,5′-dihydroxychalcone
DNP-SG: *S*-(2,4-dinitrophenyl)glutathione
GSH: reduced glutathione
MDR: multidrug resistance
MRP1 (ABCC1): multidrug resistance protein 1
P-gp (ABCB1): P-glycoprotein

## References

[B1] Ahmed-Belkacem A. Pozza A. Munoz-Martinez F. Bates S. E. Castanys S. Gamarro F. (2005). Flavonoid structure–activity studies identify 6-prenylchrysin and tectochrysin as potent and specific inhibitors of breast cancer resistance protein ABCG2. *Cancer Res.* 65 4852–4860 10.1158/0008-5472.CAN-04-1817 15930306

[B2] Allen J. D. van Loevezijn A. Lakhai J. M. van der Valk M. van Tellingen O. Reid G. (2002). Potent and specific inhibition of the breast cancer resistance protein multidrug transporter in vitro and in mouse intestine by a novel analogue of fumitremorgin C. *Mol. Cancer Ther.* 1 417–425 12477054

[B3] Allikmets R. Schriml L. M. Hutchinson A. Romano-Spica V. Dean M. (1998). A human placenta-specific ATP-binding cassette gene (ABCP) on chromosome 4q22 that is involved in multidrug resistance. *Cancer Res.* 58 5337–5339 9850061

[B4] Anderson M. E. (1985). Determination of glutathione and glutathione disulfide in biological samples. *Methods Enzymol.* 113 548–555 10.1016/S0076-6879(85)13073-9 4088074

[B5] Bakos E. Hegedüs T. Hollo Z. Welker E. Tusnàdy G. E. Zaman G. F. (1996). Membrane topology and glycosylation of the human multidrug resistance-associated protein. *J. Biol. Chem.* 271 12322–12326 10.1074/jbc.271.21.12322 8647833

[B6] Barattin R. Perrotton T. Trompier D. Lorendeau D. Di Pietro A. du Moulinet d’Hardemare A. (2010). Iodination of verapamil for a stronger induction of death, through GSH efflux, of cancer cells overexpressing MRP1. *Bioorg. Med. Chem.* 18 6265–6274 10.1016/j.bmc.2010.07.031 20691599

[B7] Battacharya S. Das A. Mallya K. Ahmad I. (2007). Maintenance of retinal stem cells by Abcg2 is regulated by notch signaling. *J. Cell Sci. *120(Pt 15) 2652–2662 10.1242/jcs.008417 17635990

[B8] Brechbuhl H. M. Gould N. Kachadourian R. Riekhof W. R. Voelker D. R. Day B. J. (2010). Glutathione transport is a unique function of the ATP-binding cassette protein ABCG2. *J. Biol. Chem.* 285 16582–16587 10.1074/jbc.M109.090506 20332504 PMC2878084

[B9] Brechbuhl H. M. Min E. Kariya C. Frederick B. Raben D. Day B. J. (2009). Select cyclopentenone prostaglandins trigger glutathione efflux and the role of ABCG2 transport. *Free Radic. Biol. Med.* 47 722–730 10.1016/j.freeradbiomed.2009.06.005 19520157 PMC2730198

[B10] Chen Q. Li W. Wan Y. Xia X. Wu Q. Chen Y. (2012). Amplified in breast cancer 1 enhances human cholangiocarcinoma growth and chemoresistance by simultaneous activation of Akt and Nrf2 pathways. *Hepatology* 55 1820–1829 10.1002/hep.25549 22213475

[B11] Chen Z. S. Robey R. W. Belinsky M. G. Shchaveleva I. Ren X. Q. Sugimoto Y. (2003). Transport of methotrexate, methotrexate polyglutamates and 17 beta-estradiol 17-(beta-D-glucuronide) by ABCG2: effects of acquired mutations at R482 on methotrexate transport. *Cancer Res.* 63 4048–4054 12874005

[B12] Cole S. P. Bhardwaj G. Gerlach J. H. Mackie J. E. Grant C. E. Almquist K. C. (1992). Overexpression of a transporter gene in a multidrug-resistant human lung cancer cell line. *Science* 258 1650–1654 10.1126/science.1360704 1360704

[B13] Cripe L. D. Uno H. Paietta E. M. Litzow M. R. Ketterling R. P. Bennett J. M. (2010). Zosuquidar, a novel modulator of P-glycoprotein, does not improve the outcome of older patients with newly diagnosed acute myeloid leukemia: a randomized, placebo-controlled trial of the Eastern Cooperative Oncology Group 3999. *Blood* 116 4077–4085 10.1182/blood-2010-04-277269 20716770 PMC2993615

[B14] Cui X. Y. Skretting G. Jing Y. Sun H. Sandset P. M. Sun L. (2013). Hypoxia influences stem cell-like properties in multidrug resistant K562 leukemic cells. *Blood Cells Mol. Dis.* 51 177–184 10.1016/j.bcmd.2013.05.003 23725749

[B15] de Boussac H. Orban T. I. Varady G. Tihanyi B. Bacquet C. Brozik A. (2012). Stimulus-induced expression of the ABCG2 multidrug transporter in HepG2 hepatocarcinoma model cells involves the ERK1/2 cascade and alternative promoters. *Biochem. Biophys. Res. Commun.* 426 172–176 10.1016/j.bbrc.2012.08.046 22922104

[B16] Deeken J. F. Robey R. W. Shukla S. Steadman K. Chakraborty A. R. Poonkuzhali B. (2009). Identification of compounds that correlate with ABCG2 transporter function in the National Cancer Institute Anticancer Drug Screen. *Mol. Pharmacol.* 76 946–956 10.1124/mol.109.056192 19633067 PMC2774997

[B17] Doyle L. A. Yang W. Abruzzo L. V. Krogmann T. Gao Y. Rishi A. K. (1998). A multidrug resistance transporter from human MCF-7 breast cancer cells. *Proc. Natl. Acad. Sci. U.S.A.* 95 15665–15670 10.1073/pnas.95.26.15665 9861027 PMC28101

[B18] Dubrovska A. Hartung A. Bouchez L. C. Walker J. R. Reddy V. A. Cho C. Y. (2012). CXCR4 activation maintains a stem cell population in tamoxifen-resistant breast cancer cells through AhR signaling. *Br. J. Cancer* 107 43–52 10.1038/bjc.2012.105 22644306 PMC3389396

[B19] Genoux-Bastide E. Lorendeau D. Nicolle E. Yahiaoui S. Magnard S. Di Pietro A. (2011). Identification of xanthones as selective killers of cancer cells overexpressing the ABC transporter MRP1. *ChemMedChem* 6 1478–1484 10.1002/cmdc.201100102 21634011

[B20] Gilani R. A. Kazi A. A. Shah P. Schech A. J. Chumsri S. Sabnis G. (2012). The importance of HER2 signaling in the tumor-initiating cell population in aromatase inhibitor-resistant breast cancer. *Breast Cancer Res. Treat.* 135 681–692 10.1007/s10549-012-2148-8 22878889

[B21] Goler-Baron V. Assaraf Y. G. (2012). Overcoming multidrug resistance via photodestruction of ABCG2-rich extracellular vesicles sequestering photosensitive chemotherapeutics. *PLoS ONE* 7:e35487 10.1371/journal.pone.0035487 PMC3329466 22530032

[B22] Hall M. D. Handley M. D. Gottesman M. M. (2009). Is resistance useless? Multidrug resistance and collateral sensitivity. *Trends Pharmacol. Sci.* 30 546–556 10.1016/j.tips.2009.07.003 19762091 PMC2774243

[B23] Hessel S. Lampen A. (2010). All-trans retinoic acid enhances the transport of phase II metabolites of benzo[a]pyrene by inducing the breast cancer resistance protein expression in Caco-2 cells. *Toxicol. Lett.* 197 151–155 10.1016/j.toxlet.2010.05.018 20562004

[B24] Hong Y. B. Kang H. J. Kwon S. Y. Kim H. J. Kwon K. Y. Cho C. H. (2010). Nuclear factor (erythroid-derived 2)-like 2 regulates drug resistance in pancreatic cancer cells. *Pancreas* 39 463–472 10.1097/MPA.0b013e3181c31314 20118824 PMC3506252

[B25] Honjo Y. Hrycyna C. A. Yan Q. W. Medina-Perez W. Y. Robey R. W. van de Laar A. (2001). Acquired mutations in the MXR/BCRP/ABCP gene alter substrate specificity in MXR/BCRP/ABCP-overexpressing cells. *Cancer Res.* 61 6635–6639 11559526

[B26] Ishikawa T. Kajimoto Y. Sun W. Nakagawa H. Inoue Y. Ikegami Y. (2013). Role of Nrf2 in cancer photodynamic therapy: regulation of human ABC transporter ABCG2. *J. Pharm. Sci.* 102 3058–3069 10.1002/jps.25563 23650051

[B27] Ito M. Kajino K. Abe M. Fujimura T. Mineki R. Ikegami T. (2013). NP-1250, an ABCG2 inhibitor, induces apoptotic cell death in mitoxantrone-resistant breast carcinoma MCF7 cells via a caspase-independent pathway. *Oncol. Rep.* 29 1492–1500 10.3892/or.2013.2249 23354844

[B28] Juliano R. L. Ling V. (1976). A surface glycoprotein modulating drug permeability in Chinese hamster ovary cell mutants. *Biochim. Biophys. Acta* 455 152–162 10.1016/0005-2736(76)90160-7 990323

[B29] Katoh M. Katoh M. (2010). Integrative genomic analyses of CXCR4: transcriptional regulation of CXCR4 based on TGFbeta, Nodal, activin signaling and POU5F1, FOXA2, FOXC2, FOXH1, SOX17, and GFI1 transcription factors. *Int. J. Oncol.* 36 415–420 10.3892/ijo_00000514 20043076

[B30] Keppler D. Leier I. Jedlitschky G. (1997). Transport of glutathione conjugates and glucuronides by the multidrug resistance proteins MRP1 and MRP2. *Biol. Chem.* 378 787–791 9377473

[B31] Kolitz J. E. George S. L. Marcucci G. Vij R. Powell B. L. Allen S. L. (2010). P-glycoprotein inhibition using valspodar (PSC-833) does not improve outcomes for patients younger than age 60 years with newly diagnosed acute myeloid leukemia: cancer and leukemia group B study 19808. *Blood* 116 1413–1421 10.1182/blood-2009-07-229492 20522709 PMC2938834

[B32] Leier I. Jedlitschki G. Buchholz U. Cole S. P. Deeley R. G. Keppler D. (1994). The MRP gene encodes an ATP-dependent export pump for leukotriene C4 and structurally related conjugates. *J. Biol. Chem.* 269 27807–27810 7961706

[B33] Li H. Gao Q. Guo L. Lu S. H. (2011). The PTEN/P13K/AKT pathway regulates stem-like cells in primary esophageal carcinoma cells. *Cancer Biol. Ther.* 11 950–958 10.4161/cbt.11.11.15531 21467840

[B34] Ludwig J. A. Szakács G. Martin S. E. Chu B. F. Cardarelli C. Sauna Z. E. (2006). Selective toxicity of NSC73306 in MDR1-positive cells as a new strategy to circumvent multidrug resistance in cancer. *Cancer Res.* 66 4808–4815 10.1158/0008-5472.CAN-05-3322 16651436 PMC1474781

[B35] Miyake K. Mickley L. Litman T. Zhan Z. Robey R. Cristensen B. (1999). Molecular cloning of cDNAs which are highly overexpressed in mitoxantrone-resistant cells: demonstration of homology to ABC transport genes. *Cancer Res.* 59 8–13 9892175

[B36] Mosmann T. (1983). Rapid colorimetric assay for cellular growth and survival: application to proliferation and cytotoxicity assays. *J. Immunol. Methods* 65 55–63 10.1016/0022-1759(83)90303-4 6606682

[B37] Nakanishi T. Ross D. D. (2012). Breast cancer resistance protein (BCRP/ABCG2): its role in multidrug resistance and regulation of its gene expression. *Chin. J. Cancer* 31 73–99 10.5732/cjc.011.10320 22098950 PMC3777471

[B38] Ozvegy C. Litman T. Szakacs G. Nagy Z. Bates S. E. Varadi A. (2001). Functional characterization of the human multidrug transporter, ABCG2, expressed in insect cells. *Biochem. Biophys. Res. Commun.* 285 111–117 10.1006/bbrc.2001.5130 11437380

[B39] Ozvegy-Laczka C. Köblös G. Sarkadi B. Varadi A. (2005). Single amino acid (482) variants of the ABCG2 multidrug transporter: major differences in transport capacity and substrate recognition. *Biochim. Biophys. Acta* 1668 53–63 10.1016/j.bbamem.2004.11.005 15670731

[B40] Perrotton T. Trompier D. Chang X-B. Di Pietro A. Baubichon-Cortay H. (2007). S- and R-verapamil differentially modulate the multidrug resistance protein MRP1. *J. Biol. Chem.* 282 31542–31548 10.1074/jbc.M703964200 17646169

[B41] Pluchino K. M. Hall M. D. Goldsborough A. S. Callaghan R. Gottesman M. M. (2012). Collateral sensitivity as a strategy against cancer multidrug resistance. *Drug Resist. Updat.* 15 98–105 10.1016/j.drup.2012.03.002 22483810 PMC3348266

[B42] Robey R. W. Honjo Y. Morisaki K. Nadjem T. A. Runge S. Risbood M. (2003). Mutations at amino-acid 482 in the ABCG2 gene affect substrate and antagonist specificity. *Br. J. Cancer* 89 1971–1978 10.1038/sj.bjc.6601370 14612912 PMC2394461

[B43] Robey R. W. Massey P. R. Amiri-Kordestani L. Bates S. E. (2010). ABC transporters: unvalidated therapeutic targets in cancer and the CNS. *Anticancer Agents Med. Chem.* 10 625–633 10.2174/187152010794473957 21189132 PMC3119022

[B44] Shelton P. Jaiswal A. K. (2013). The transcription factor NF-E2-related factor 2 (Nrf2): a protooncogene? *FASEB J.* 27 414–423 10.1096/fj.12-217257 23109674 PMC3545532

[B45] Shen S. Callaghan D. Juzwik C. Xiong H. Huang P. Zhang W. (2010). ABCG2 reduces ROS-mediated toxicity and inflammation: a potential role in Alzheimer’s disease. *J. Neurochem.* 114 1590–1604 10.1111/j.1471-4159.2010.06887.x 20626554

[B46] Singh A. Wu H. Zhang P. Happel C. Ma. J. Biswal S. (2010). Expression of ABCG2 (BCRP) is regulated by Nrf2 in cancer cells that confers side population and chemoresistance phenotype. *Mol. Cancer Ther.* 9 2365–2376 10.1158/1535-7163.MCT-10-0108 20682644 PMC2955865

[B47] Singh R. R. Kunkalla K. Qu C. Schlette E. Neelapu S. S. Samaniego F. (2011). Abcg2 is a direct transcriptional target of hedgehog signaling and involved in stroma-induced drug tolerance in diffuse large B-cell lymphoma. *Oncogene* 30 4874–4886 10.1038/onc.2011.195 21625222 PMC3165099

[B48] Suzuki M. Suzuki H. Sugimoto Y. Sugiyama Y. (2003). ABCG2 transports sulfated conjugates of steroids and xenobiotics. *J. Biol. Chem.* 278 22644–22649 10.1074/jbc.M212399200 12682043

[B49] Szakacs G. Paterson J. K. Ludwig J. A. Booth-Genthe C. Gottesman M. M. (2006). Targeting multidrug resistance in cancer. *Nat. Rev. Drug Discov.* 5 219–234 10.1038/nrd1984 16518375

[B50] Szakács G. Varadi A. Ozvegy-Laczka C. Sarkadi B. (2008). The role of ABC transporters in drug absorption, distribution, metabolism, excretion and toxicity (ADME-tox). *Drug Discov. Today* 13 379–393 10.1016/j.drudis.2007.12.010 18468555

[B51] Telbisz A. Hegedus C. Ozvegy-Laczka C. Goda K. Varady G. Takats Z. (2012). Antibody binding shift assay for rapid screening of drug interactions with the human ABCG2 multidrug transporter. *Eur. J. Pharm. Sci.* 45 101–109 10.1016/j.ejps.2011.10.021 22115866

[B52] Telbisz A. Müller M. Ozvegy-Laczka C. Homolya L. Szente L. Varadi A. (2007). Membrane cholesterol selectively modulates the activity of the human ABCG2 multidrug transporter. *Biochim. Biophys. Acta* 1768 2698–2713 10.1016/j.bbamem.2007.06.026 17662239

[B53] Tietze F. (1969). Enzymic method for quantitative determination of nanogram amounts of total and oxidized glutathione: applications to mammalian blood and other tissues. *Anal. Biochem.* 27 502–522 10.1016/0003-2697(69)90064-5 4388022

[B54] Trompier D. Chang X. B. Barattin R. du Moulinet d’Hardemare A. Di Pietro A. Baubichon-Cortay H. (2004). Verapamil and its derivative trigger apoptosis through glutathione extrusion by multidrug resistance protein MRP1. *Cancer Res.* 64 4950–4956 10.1158/0008-5472.CAN-04-0143 15256468

[B55] Türk D. Hall M. D. Chu B. F. Ludwig J. A. Fales H. M. Gottesman M. M. (2009). Identification of compounds selectively killing multidrug-resistant cancer cells. *Cancer Res.* 69 8293–8301 10.1158/0008-5472.CAN-09-2422 19843850 PMC2783730

[B56] Usongo M. Farookhi R. (2012). β-Catenin/Tcf-signaling appears to establish the murine ovarian surface epithelium (OSE) and remains active in selected postnatal OSE cells. *BMC Dev. Biol.* 12:17 10.1186/1471-213X-12-17 PMC3465187 22682531

[B57] Valdameri G. Gauthier C. Terreux R. Kachadourian R. Day B. J. Winnischofer S. M. (2012). Investigation of chalcones as selective inhibitors of the breast cancer resistance protein: critical role of methoxylation in both inhibition potency and cytotoxicity. *J. Med. Chem.* 55 3193–3200 10.1021/jm2016528 22449016 PMC3983950

[B58] Wang X. Q. Ongkeko W. M. Chen L. Yang Z. F. Lu P. Chen K. K. (2010). Octamer 4 (Oct4) mediates chemotherapeutic drug resistance in liver cancer cells through a potential Oct4-AKT-ATP-binding cassette G2 pathway. *Hepatology* 52 528–539 10.1002/hep.23692 20683952

[B59] Zhang M. Mathur A. Zhang Y. Xi S. Atay S. Hong J. A. (2012). Mithramycin represses basal and cigarette smoke-induced expression of ABCG2 and inhibits stem cell signaling in lung and esophageal cancer cells. *Cancer Res.* 72 4178–4192 10.1158/0008-5472.CAN-11-3983 22751465 PMC6261440

[B60] Zhu M. M. Tong J. L. Xu Q. Nie F. Xu X. T. Xia S. D. (2012). Increased JNK1 signaling pathway is responsible for ABCG2-mediated multidrug resistance in human colon cancer. *PLoS ONE* 7:e41763 10.1371/journal.pone.0041763 PMC3411563 22870247

